# Transcervical video-assisted mediastinoscopy for resection of an ectopic parathyroid adenoma in the mediastinum: a case report

**DOI:** 10.3389/fsurg.2026.1803289

**Published:** 2026-04-30

**Authors:** Zhanfeng Su, He Wang, Lei Yang, Jixian Liu

**Affiliations:** 1 Department of Thoracic Surgery, Peking University Shenzhen Hospital, Shenzhen, China; 2 Guangdong Medical University, Zhanjiang, China

**Keywords:** 99mTc-sestamibi (99mTc-MIBI) SPECT/CT, intraoperative parathyroid hormone (IOPTH), mediastinal ectopic parathyroid adenoma (MEPA), primary hyperparathyroidism (pHPT), video-assisted mediastinoscopy (VAM)

## Abstract

**Background:**

Mediastinal ectopic parathyroid adenoma (MEPA) presents significant difficulties in both preoperative localization and surgical management due to its deep mediastinal location and close relationship to critical surrounding structures. Traditional surgical approaches, such as median sternotomy or thoracotomy, are often necessary but are associated with considerable surgical morbidity. Currently, minimally invasive options for resecting deeply located mediastinal parathyroid lesions are limited and remain technically challenging.

**Case presentation:**

We report a rare case of primary hyperparathyroidism (PHPT) in a 37-year-old male presenting with persistent hypercalcemia and elevated parathyroid hormone (PTH) levels, caused by a mediastinal ectopic parathyroid adenoma located in the aortopulmonary (AP) window. While initial imaging modalities failed to localize the lesion, it was successfully identified using 99mTc-sestamibi (99mTc-MIBI) SPECT/CT. The patient underwent transcervical video-assisted mediastinoscopy (VAM), and complete resection of the adenoma was confirmed via intraoperative parathyroid hormone (ioPTH) monitoring. The postoperative course was marked by transient hypocalcemia and rebound PTH elevation attributed to hungry bone syndrome. Following titrated oral calcium and calcitriol supplementation, sustained biochemical remission was achieved at the 3-month follow-up.

**Conclusions:**

This case highlights the diagnostic challenges of mediastinal ectopic parathyroid adenomas, advocating for 99mTc-MIBI SPECT/CT for definitive localization when cervical imaging is inconclusive. Transcervical VAM offers a viable and advantageous, minimally invasive approach for accessing carefully selected lesions in the AP window, with ioPTH monitoring essential to confirm successful resection. Additionally, careful monitoring for postoperative hungry bone syndrome is necessary to ensure sustained biochemical remission.

## Introduction

Solitary ectopic parathyroid adenomas cause approximately 80% of primary hyperparathyroidism (PHPT) (1, 2). While typically cervical, 15%–20% of lesions are ectopic, predominantly mediastinal (3). The etiology of mediastinal ectopic parathyroid adenoma (MEPA) results from aberrant migration of the pharyngeal pouches. Incomplete thymic separation facilitates the descent of thymic tissue into deep structures such as the aortopulmonary (AP) window (4, 5). Intricate vascular proximity makes localization and resection in the AP window difficult (6). Traditional sternotomy or thoracotomy is invasive, carrying significant surgical risk.

## Case presentation

### Patient history and preoperative evaluation

**Figure 1:**
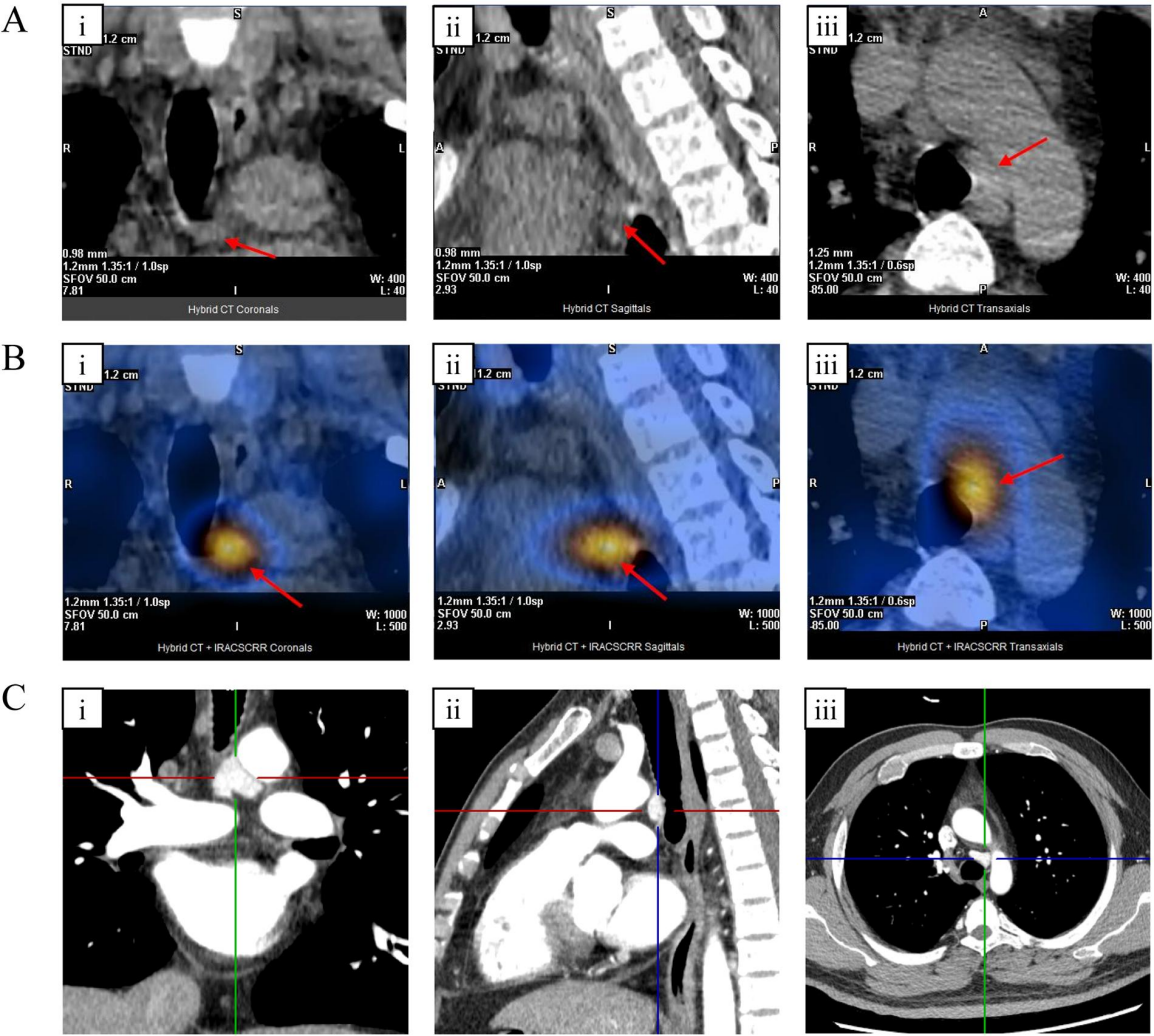
**(A)** Parathyroid SPECT/CT (CT component): coronal **(i)**, sagittal **(ii)**, and axial **(iii)** views demonstrating a solid nodule with well-defined borders and slightly heterogeneous density in the superior mediastinum, located anterior to the left side of the trachea and inferior to the aortic arch. **(B)** 99mTc-MIBI SPECT/CT (Fusion): Corresponding functional images **(i–iii)** revealing focal increased radiotracer uptake in the nodule, indicating hyperfunctioning parathyroid tissue. **(C)** Coronal **(i)**, sagittal **(ii)**, and axial **(iii)** multiplanar reconstruction views identifying a triangular soft-tissue mass (approx. 21 mm × 15 mm × 20 mm) in the same region.

A 37-year-old male was admitted with a chief complaint of right-sided chest pain accompanied by bilateral lower extremity weakness. One month prior to admission, laboratory evaluation revealed significant hypercalcemia (serum calcium: 3.02 mmol/L; reference range: 2.10–2.55 mmol/L) and markedly elevated intact parathyroid hormone (serum PTH: 911.90 pg/mL; reference range: 12.26–87.70 pg/mL). The serum alkaline phosphatase (ALP) was also markedly elevated to 404 U/L (reference range: 45–125 U/L), reflecting intense bone turnover. Dual-energy x-ray absorptiometry (DEXA) revealed significantly reduced bone mineral density compared to age-matched peers, with Z-scores of −3.0 at the lumbar spine (L1-L4), −2.2 at the left femoral neck, and −2.3 at the total left hip. The patient denied respiratory symptoms or fever. Significant history included recurrent bilateral nephrolithiasis for 5 years. Prior neck surgery, trauma, and familial endocrine disorders were denied. Physical examination was largely unremarkable, with no palpable neck masses or localized bone tenderness. Non-contrast chest computed tomography (CT) identified multiple osteolytic lesions involving the ribs and thoracic vertebrae, alongside bilateral renal calculi, raising a strong suspicion for advanced PHPT. With cervical ultrasound and neck CT being non-diagnostic, 99mTc-MIBI SPECT/CT identified a 19 × 9 mm nodule in the mediastinal AP window (Figure 1A) exhibiting focal uptake (Figure 1B). Contrast-enhanced CT subsequently delineated the lesion's critical relationship with the aortic arch and trachea (Figure 1C). Normal values for thyroid function, aldosterone, IGF-1, prolactin, ACTH, and renin ruled out multiple endocrine neoplasia (MEN) types 1 or 2a. Given the negative family history and negative biochemical screening, routine genetic testing for MEN mutations was not pursued at this stage.

### Surgical resection and pathological findings

**Figure 2:**
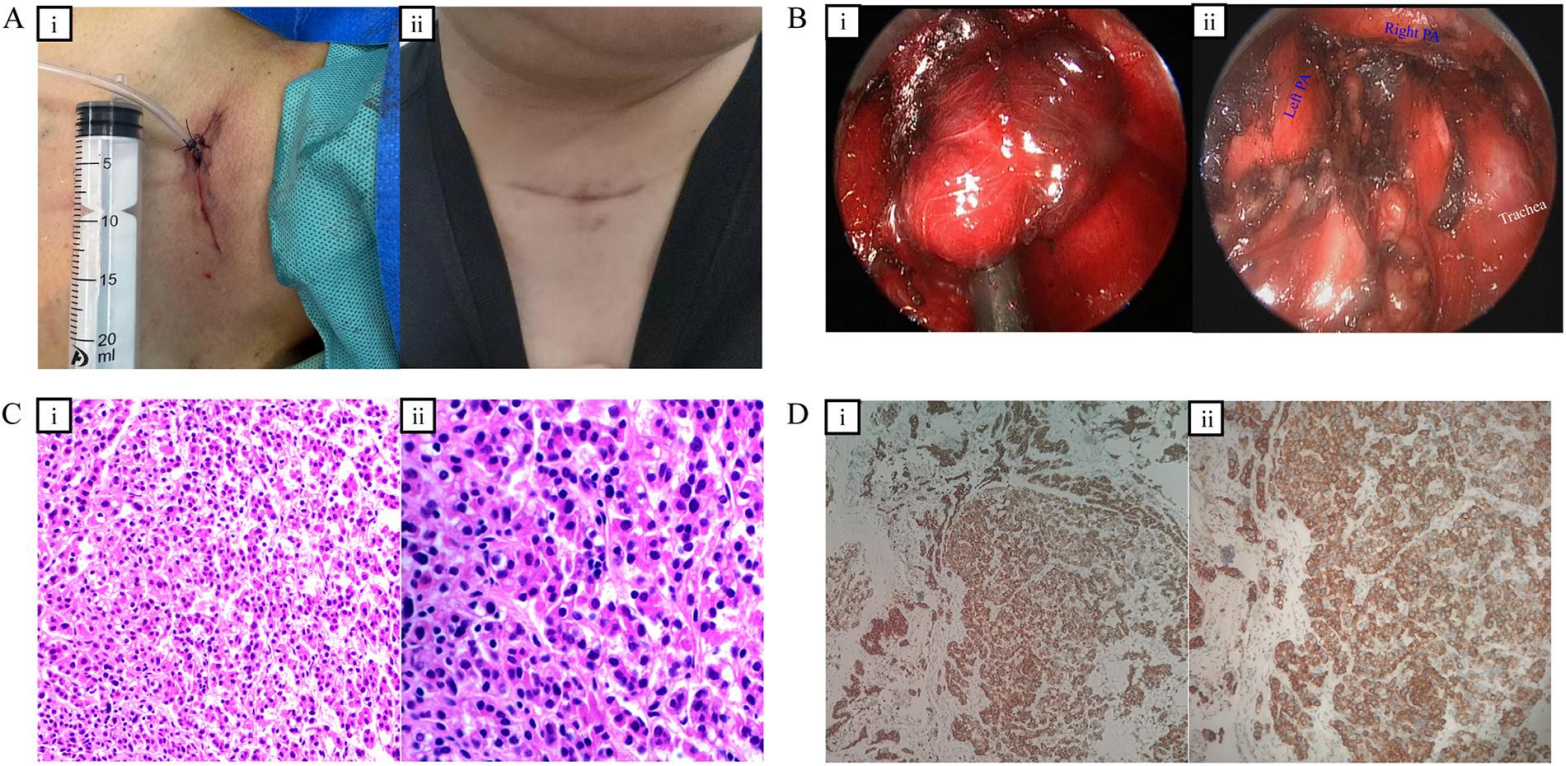
**(A)** Transcervical approach: **(i)** A 3-cm incision made at the suprasternal notch; **(ii)** Postoperative view demonstrating a well-healed incision. **(B)** Intraoperative mediastinoscopic views: **(i)** The ectopic adenoma identified beneath the aortic arch; **(ii)** The post-resection tumor bed, revealing the pulmonary arteries anteroinferiorly and the bronchus posteriorly. **(C)** Histopathological examination (H&E staining): **(i)** Low-magnification view demonstrating a nested pattern of predominantly eosinophilic cells admixed with scattered clear cells; **(ii)** High-magnification view revealing round, hyperchromatic nuclei with visible nucleoli and rare mitotic figures. **(D)** Immunohistochemical staining: **(i)** Low-magnification and **(ii)** high-magnification views demonstrating diffuse cytoplasmic positivity for parathyroid hormone (PTH), confirming the parathyroid origin of the tumor.

Prior to surgery, the patient underwent medical optimization for hypercalcemia utilizing zoledronic acid, salmon calcitonin, and furosemide diuresis. Immediate preoperative biochemical assessment revealed a serum PTH of 860.02 pg/mL and a serum calcium level normalized to 2.55 mmol/L. The procedure utilized a transcervical VAM approach under general anesthesia, preserving double-lung ventilation. A 3-cm curvilinear incision was made at the suprasternal notch (Figure 2A). Following blunt dissection into the pretracheal space, the mediastinoscope was introduced, and the dissection plane was advanced caudally along the trachea to the level of the carina. The tumor was visualized deep within the AP window, inferior to the aortic arch (Figure 2B). Meticulous dissection was performed to ensure preservation of the recurrent laryngeal nerve, the aortic arch, and the pulmonary artery.

Curative resection was verified via intraoperative parathyroid hormone (ioPTH) monitoring to satisfy the Miami criteria. Blood samples were drawn at the pre-excision baseline, and sequentially at 5 min and 24 min post-excision. The serum PTH level plummeted from a baseline of 860.02 pg/mL to 82.04 pg/mL at the 5-minute post-excision mark. A subsequent measurement taken 19 min after the first post-excision sample demonstrated a further decline to 66.95 pg/mL. This rapid decline of over 90% at the initial 5-minute evaluation robustly fulfilled and exceeded the Miami criterion (which requires a > 50% drop at 10 min), reliably confirming the complete extirpation of the hyperfunctioning tissue before terminating the procedure. Intraoperative serum calcium was 2.52 mmol/L. The total operative duration was 200 min, with minimal estimated blood loss (20 mL). Macroscopic examination of the resected specimen revealed an encapsulated, homogeneous, solid red-brown nodule weighing 2.7 g and measuring 2.8 × 1.8 × 0.7 cm. Microscopically, the tumor parenchyma was composed of nests and sheets of cells interspersed with a rich capillary network. The cellular composition was predominantly oxyphil cells, characterized by abundant eosinophilic cytoplasm and clear borders, admixed with scattered chief cells. The nuclei were centrally located, round, and hyperchromatic with visible nucleoli. Crucially, there were rare mitotic figures, and no evidence of capsular invasion, vascular invasion, or thick fibrous bands was observed (Figure 2C). Immunohistochemical (IHC) analysis demonstrated strong, diffuse cytoplasmic positivity for parathyroid hormone (PTH) (Figure 2D), confirming the parathyroid origin of the tumor. The neuroendocrine nature of the lesion was supported by positive staining for Chromogranin A (CgA), despite negative synaptophysin (SYN) expression. Furthermore, pan-cytokeratin (CK-pan) was positive, confirming its epithelial lineage. Notably, the Ki-67 proliferation index was exceptionally low at approximately 2%. This low proliferative activity, combined with the complete absence of invasive morphological features, definitively excluded parathyroid carcinoma and robustly supported the diagnosis of a benign ectopic parathyroid adenoma.

### Postoperative management and follow-up

**Figure 3:**
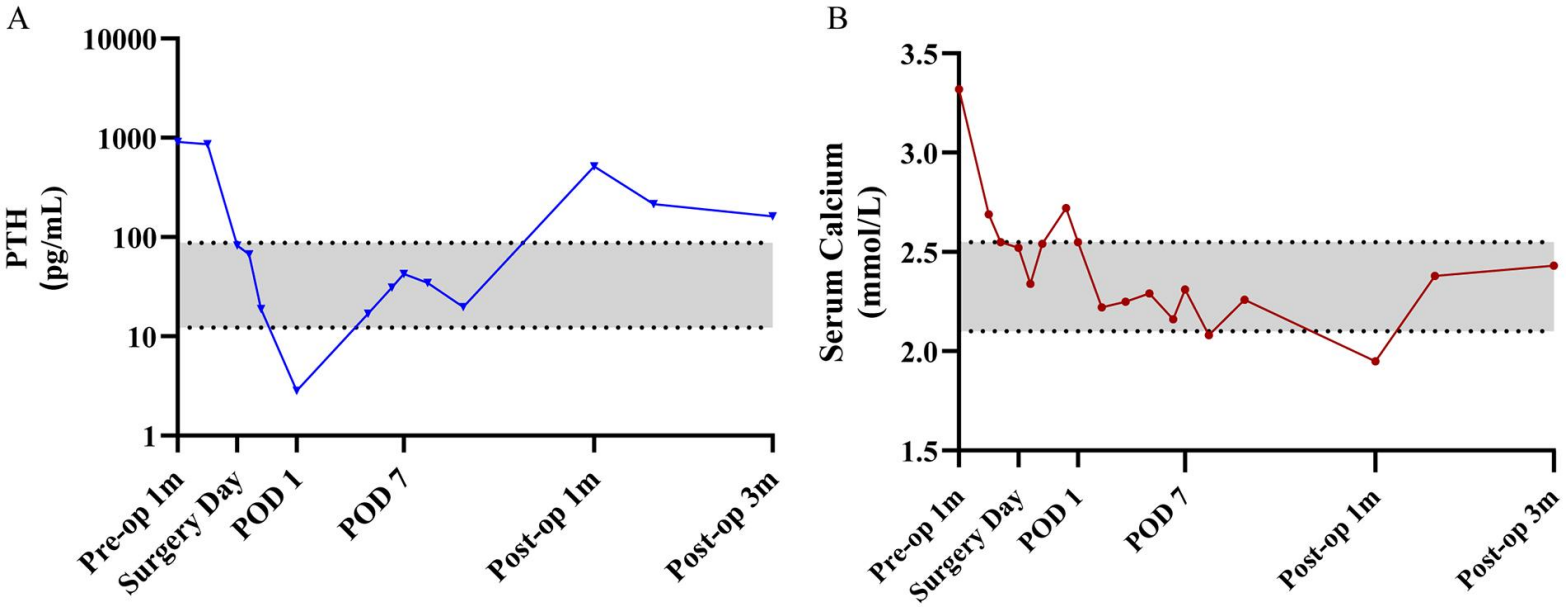
Perioperative trends of serum PTH and calcium levels are shown. Gray shaded regions represent the institutional reference ranges for PTH (12.26–87.70 pg/mL) and serum calcium (2.10–2.55 mmol/L). **(A)** PTH levels exhibited a precipitous drop immediately following surgery, followed by a transient rebound at 1 month; by the 3-month follow-up, levels showed a gradual decline but remained slightly above the upper limit of the normal range. **(B)** Serum calcium levels dipped below the normal range at 1 month postoperatively (hypocalcemia) but were successfully restored to the normal range by 3 months after oral supplementation. Pre-op 1 m, 1 month prior to surgery; POD 1/7, postoperative day 1 and 7; Post-op 1 m/3 m, 1 and 3 months after surgery.

The postoperative course was uneventful. Hypocalcemia prophylaxis was initiated with a tapered regimen of intravenous calcium gluconate and oral calcitriol, guided by serial serum calcium and PTH measurements. As serum calcium levels stabilized, intravenous supplementation was weaned in favor of oral maintenance. Clinically, the patient reported complete resolution of chest pain and significant recovery of lower extremity motor function. At the time of discharge, biochemical profiles remained stable within normal limits (serum calcium: 2.26 mmol/L; serum PTH: 46.21 pg/mL), and the patient was discharged without complications. Upon discharge, the patient was prescribed oral calcium carbonate (0.6 g, t.i.d.) and calcitriol (0.25 μg, b.i.d.). At the one-month postoperative follow-up, serum PTH was 513.93 pg/mL and calcium was 1.95 mmol/L. Notably, the patient remained asymptomatic, reporting no signs of hypocalcemia such as perioral numbness, digital paresthesia, or tetany. Consequently, the regimen was adjusted: calcium carbonate was increased to 1.2 g b.i.d., while calcitriol was maintained at 0.25 μg b.i.d. Two weeks after this adjustment, serum calcium normalized to 2.26 mmol/L, and PTH levels significantly decreased to 214.06 pg/mL. At the three-month follow-up, serum calcium remained within the normal range, and PTH further declined to 161.25 pg/mL (Figure 3). Concurrently, the serum ALP level decreased significantly to 137 U/L, indicating a gradual stabilization of bone turnover.

### Timeline

**Figure 4:**
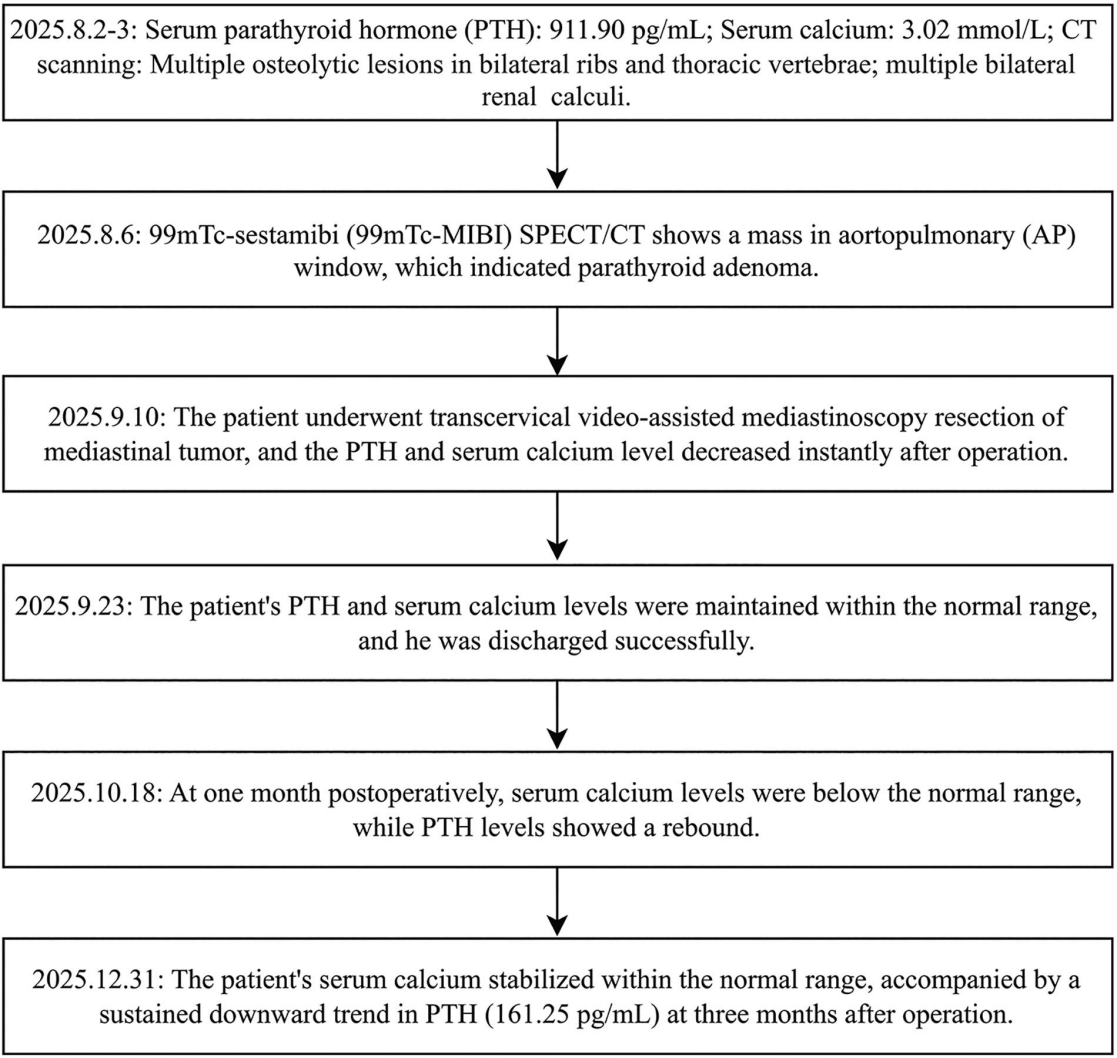
The timeline of clinical interventions of this patient.

The timeline of clinical interventions for this patient is shown in Figure 4.

## Discussion

Surgical excision remains the definitive curative treatment for MEPA causing PHPT (7). Historically, median sternotomy or thoracotomy served as the standard approach. However, these open procedures are associated with significant morbidity, including extensive tissue trauma, prolonged hospitalization, and increased risks of bleeding and neurovascular injury (8). While video- and robot-assisted thoracoscopic surgery (VATS/RATS) are minimally invasive, they necessitate transthoracic access, single-lung ventilation, and chest tubes, introducing pulmonary risks (9, 10). In contrast, VAM creates a pretracheal corridor that spares the pleural cavity, eliminating single-lung ventilation. This provides distinct advantages for AP window or superior thymic pole lesions, which are anatomically difficult to visualize via lateral thoracoscopy (11, 12).

Accurate preoperative localization is essential for the success of minimally invasive parathyroidectomy. In this patient, conventional cervical ultrasonography failed to identify the lesion, a common limitation when adenomas are located in the mediastinum. This highlights the importance of multimodal imaging. The use of 99mTc-MIBI SPECT/CT fusion imaging proved critical in our case, offering precise anatomical localization relative to the aortic arch and thereby guiding the surgical approach. Real-time biochemical confirmation via ioPTH monitoring is indispensable for verifying the complete excision of occult lesions (13), particularly in anatomically complex regions like the AP window. The classic Miami criterion dictates a > 50% decline in ioPTH from the highest pre-excision baseline at 10 min post-excision to confirm curative surgery. In our case, the ioPTH plummeted by >90% (from 860.02 to 82.04 pg/mL) at 5 min post-excision, which fulfilled the Miami criteria. This precipitous drop provided immediate confidence in the complete removal of the hyperfunctioning tissue, allowing for the termination of the procedure without unnecessary further exploration. Although VAM is a minimally invasive approach, the 200-minute operative duration in this case reflects the meticulous dissection required within the narrow AP window to avoid injuring critical adjacent structures (aortic arch, pulmonary artery, and recurrent laryngeal nerve). Furthermore, this timeframe inherently encompasses the intraoperative waiting periods for frozen section analysis and sequential ioPTH results. Despite its advantages, VAM possesses inherent technical limitations. The narrow pretracheal working space makes it challenging for exceptionally large adenomas or in patients with severe mediastinal adhesions from prior anterior neck or thoracic surgeries. Furthermore, dissecting deep within the AP window requires advanced endoscopic skills and carries a steep learning curve. Therefore, stringent patient selection is paramount. Ideal candidates are those with a solitary, precisely localized lesion via multimodal imaging (e.g., SPECT/CT), lacking prior extensive mediastinal interventions, and where the adenoma size is amenable to transcervical extraction.

The multiple osteolytic lesions and severely reduced bone mineral density (Z-score −3.0 at the lumbar spine) observed in this patient represent osteitis fibrosa cystica (brown tumors), the classic skeletal manifestation of advanced PHPT. These lesions consist of highly vascularized fibrous tissue replacing normal bone due to relentless osteoclastic resorption driven by persistently elevated PTH. Postoperatively, managing hungry bone syndrome (HBS) is a critical consideration for patients with long-standing PHPT and elevated preoperative PTH levels. The abrupt cessation of PTH can result in rapid bone remineralization and severe hypocalcemia. The case underscores the importance of a dynamic, stepwise protocol that transitions from intravenous to oral calcium supplementation, guided by serial serum calcium monitoring. This proactive approach effectively reduces the risk of symptomatic hypocalcemia and promotes a smooth recovery (14). At the one-month follow-up, a noteworthy rebound elevation in PTH (513.93 pg/mL) was observed. We mechanistically attribute this to a severe manifestation of HBS rather than the presence of residual hyperfunctional tissue. Crucially, residual autonomous parathyroid tissue would present with concomitant hypercalcemia alongside elevated PTH. In contrast, our patient exhibited profound hypocalcemia (1.95 mmol/L) during this PTH spike. The sudden surgical removal of the MEPA abruptly halted osteoclastic bone resorption. Consequently, uncoupled, markedly increased osteoblastic activity created a substantial “calcium sink” for the remineralization of the extensive osteolytic lesions. This profound hypocalcemic state reflexively stimulated the calcium-sensing receptors of the remaining, previously suppressed normal parathyroid glands, triggering an appropriate, compensatory secondary surge in PTH secretion. Following aggressive upward titration of oral calcium and calcitriol, the serum calcium normalized, and the PTH levels steadily trended downwards (161.25 pg/mL at 3 months). This longitudinal biochemical dynamic further substantiates the physiological nature of the rebound and clinically excludes residual disease.

## Conclusion

In cases of PHPT characterized by hypercalcemia and non-diagnostic cervical imaging, clinical suspicion must remain high for ectopic mediastinal pathology. This case highlights the critical utility of multimodal imaging protocols—specifically the incorporation of 99mTc-MIBI SPECT/CT fusion imaging—in facilitating the precise preoperative localization of occult lesions. For deep mediastinal adenomas, transcervical VAM represents a feasible, effective, and safe surgical modality. By providing adequate visualization of neurovascular structures within the AP window without breaching the pleural cavity, it minimizes surgical morbidity and reduces the risk of postoperative complications. Intraoperative PTH monitoring remains essential to confirm immediate biochemical remission. Postoperatively, vigilance for hungry bone syndrome is required in patients with significant skeletal involvement; long-term follow-up with titration of calcium and vitamin D is necessary to manage rebound PTH elevation.

## Patient perspective

I had been struggling with chest pain and weak legs, so I am incredibly happy that this surgery resolved my issues. The doctors took such good care of me, and I am truly grateful to the whole team for my recovery.

## Data Availability

The original contributions presented in the study are included in the article/Supplementary Material, further inquiries can be directed to the corresponding author/s.
